# Efficacy of the Ovesco Clip for Closure of Endoscope Related Perforations

**DOI:** 10.1155/2016/9371878

**Published:** 2016-05-16

**Authors:** Phonthep Angsuwatcharakon, Piyapan Prueksapanich, Pradermchai Kongkam, Thawee Rattanachu-ek, Jaksin Sottisuporn, Rungsun Rerknimitr

**Affiliations:** ^1^Department of Medicine, Faculty of Medicine, King Chulalongkorn Memorial Hospital, Chulalongkorn University, Bangkok 10330, Thailand; ^2^Department of Anatomy, Faculty of Medicine, King Chulalongkorn Memorial Hospital, Chulalongkorn University, Bangkok 10330, Thailand; ^3^Center of Excellence, Laparoscopic Endoscopic Surgery, Department of Surgery, Rajavithi Hospital, Bangkok 10400, Thailand; ^4^NKC Institute of Gastroenterology and Hepatology, Faculty of Medicine, Prince of Songkla University, Songkhla 90110, Thailand

## Abstract

*Aim*. To study the efficacy and other treatment outcomes of Ovesco clip closure of iatrogenic perforation.* Methods*. Retrospective study from 3 tertiary-care hospitals in Thailand. Patients with iatrogenic perforation who underwent immediate endoscopic closure by Ovesco clip were included. Patients' demographic data, perforation size, number of Ovesco clips used, fasting day, length of hospital stay, success rates, and complication rate were recorded. Technical success was defined as closure achievement during endoscopic procedure and clinical success was defined as the patient can be discharged without the need of additional surgical or radiological intervention.* Results*. There were 6 iatrogenic perforations in 2 male and 4 female patients. The median age was 59 years (range 39–78 years). The locations of perforation were 5 duodenal walls and 1 rectosigmoid junction. The median perforation size was 13 mm (range 10–40 mm). The technical success was 100% and the clinical success was 83.3%. The success rates per locations were 100% in colon and 80% in duodenum, respectively. The median fasting time was 5 days (range 1–10 days) and the median length of hospital stay was 10 days (range 2–22 days). There was no mortality in any.* Conclusion*. Ovesco clip seems to be an effective and safe tool for a closure of iatrogenic perforation.

## 1. Introduction

Iatrogenic perforation during gastrointestinal endoscopy is generally needed surgical management [1] because surgery provides a complete closure as well as an opportunity for a clearance of the spillage content. However, surgical interventions carry significant morbidity and mortality especially in patients with comorbid illness [2, 3]. Early detection and closure are the key factors for a favorable outcome [4, 5]. Immediate closure during endoscopy may reduce the risk of further spillage of gastrointestinal content [6, 7] and should be the optimal time-point to carry; however, the reliable device is needed for this purpose.

Ovesco clip (Tübingen, Germany), an over-the-scope clip (OTSC), is made from nitinol which is biocompatible and has self-memory shape. The clip is mounted on an applicator cap. When using, the cap will be affixed to the tip of the scope. There is a threat connecting between the clip and hand wheel. The manner of deployment is similar to variceal band ligation. Ovesco has been approved in Europe since 2009 and in the US since 2010 [8]. Ovesco provides the secure closure strength as tight as hand suture in ex vivo porcine model [9] because it can grasp all layers of visceral wall and this in turn leads to a full thickness healing [10]. Among leakage indications of Ovesco, acute perforation has the most success rate when compared with other postoperative leakages or fistulas because acute perforation contains fresh ulcer edge with less fibrosis [11, 12]. We aim to evaluate the performance of OTSC for perforation closure in the tertiary-care hospitals in Thailand.

## 2. Methods

Endoscopy database from 3 tertiary-care hospitals in Thailand including King Chulalongkorn Memorial Hospital, Rajavithi Hospital, and Songklanagarind Hospital were retrospectively reviewed. Patients with iatrogenic perforation who received endoscopic closure by Ovesco clip were included in this study.

The procedures of endoscopic perforation closure were as follows. Once perforation was detected during endoscopy procedure, the insufflation was switched from air to CO_2_ where applicable, broad-spectrum antibiotic was administered intravenously, and the vital signs were closely monitored. The Ovesco clip cap was mounted at the tip of the end-viewing scope; the thread connected between the cap and control wheel was inserted through the accessory channel. The twin-grasper was used only when the perforation edges were apart. During closure, the perforation edge was suctioned into an applicator cap and then the clip was deployed. Enterogram using water soluble contrast media was performed to confirm the complete closure. After procedure, patients were admitted to the hospital and closely observed. Duration of fasting depended on endoscopists' discretion. Technical success is defined as successful closure of perforation confirmed by enterogram and clinical success is defined as the patient can be discharged without the need of surgical or radiological intervention.

## 3. Results

**Figure 1:**
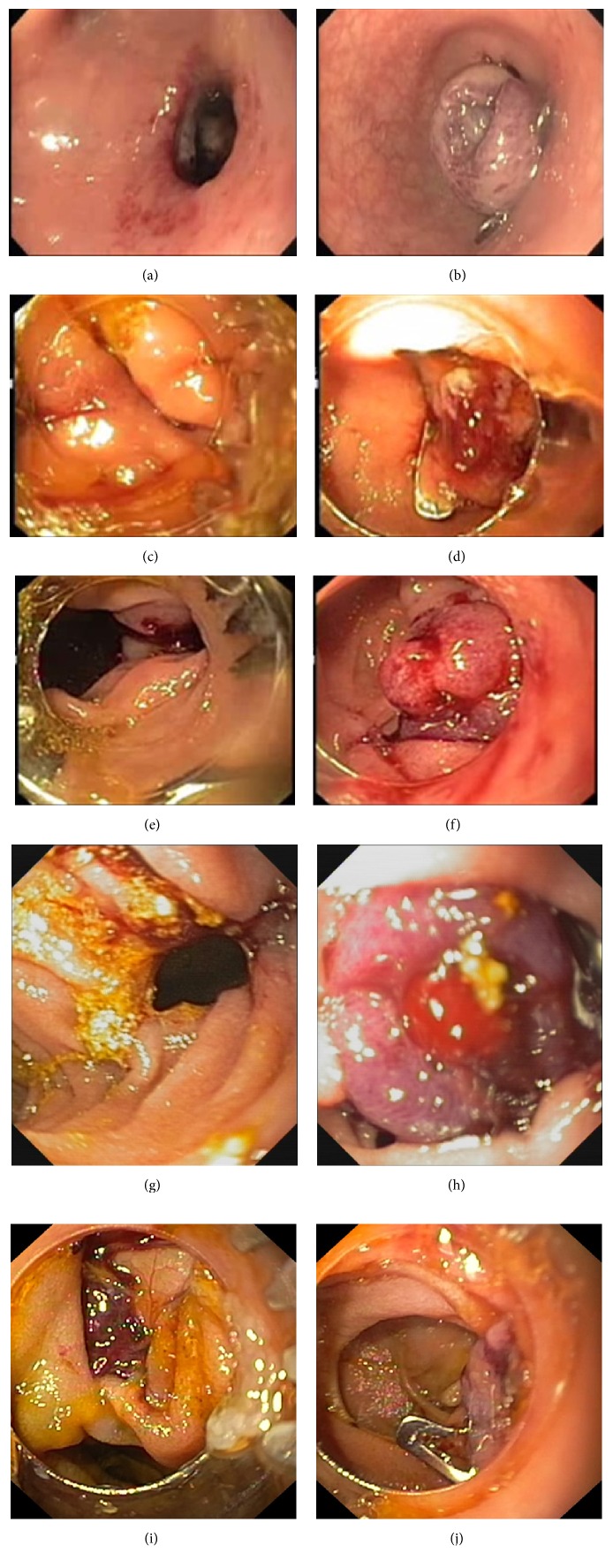
Endoscopic pictures of patients with successful closure by one Ovesco clip. (a) Rectosigmoid perforation; (b) after Ovesco clip closure of rectosigmoid perforation; (c) medial duodenal wall perforation; (d) after Ovesco clip closure of medial duodenal wall perforation; (e, g, i) lateral duodenal wall perforations; (f, h, j) after Ovesco clip closure of lateral duodenal wall perforations.

There were 6 iatrogenic perforations (Table 1) caused by colonoscope (*n* = 1), needle-knife creation of choledochoduodenostomy tract (*n* = 1), and duodenoscope (*n* = 4). Locations of perforation were rectosigmoid colon (1), medial duodenal wall (1), and lateral duodenal wall (4). There were 2 male and 4 female patients with median age of 59 years (range 39–78 years). The median size of perforation was 13 mm (range 10–40 mm). Five patients (83.3%) required 1 Ovesco clip (Figure 1) and one patient needed 2 Ovesco clips and 1 rubber band to achieve a complete closure. Technical success and clinical success rates of endoscopic closure were 100% and 83.3%, respectively. The median fasting time was 5 days (range 1–10 days) and the median length of stay was 10 days (range 2–22 days).

**Figure 2:**
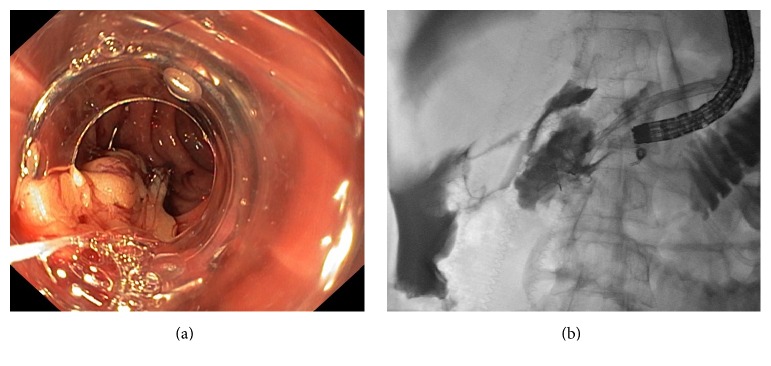
A case with 40 mm perforation at duodenal wall. (a) Two Ovesco clips with omental patch were applied; (b) enterogram revealed persistent contrast leakage indicating an incomplete closure.

**Figure 3:**
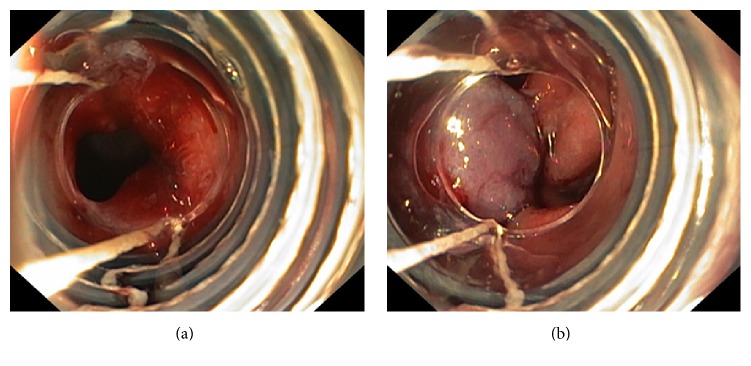
Rescue band ligation after 2 Ovesco clips' application. (a) A residual perforation was detected; (b) successful closure with an additional band ligation.

One patient was diagnosed with chronic pancreatitis with pancreatic duct obstruction from stone and stricture. He had been placed with a pancreatic stent for 3 months and was scheduled for ERCP and stent exchange. A 40 mm perforation was detected during duodenoscope insertion. After the application of two Ovesco clips with omental patch, the perforation could not be completely closed as confirmed by the presence of contrast leakage during enterogram (Figure 2). One rescue band was attempted and successfully closed the perforation (Figure 3). He had no fever or abdominal pain and could resume oral intake by day 8 after closure. The intraperitoneally misplaced Ovesco clips were incidentally found by computed tomography on day 9. After surgical consultation, the patient underwent laparoscopy on day 11 which found that 2 Ovesco clips were dislodged intraperitoneally and wrapped by the omentum; laparoscopic removal of 2 Ovesco clips without need of duodenal repair was done. He was discharged on day 12, the next day after laparoscopy. He underwent ERCP at another 3 months for pancreatic stent exchange without any complication. In another patient with rectosigmoid perforation, a repeated colonoscopy was successful within the next month after a successful endoscopic closure by an Ovesco clip. In a patient who underwent an EUS-guided choledochoduodenostomy for failed biliary cannulation, a percutaneous transhepatic biliary drainage was performed immediately after closure, and the second ERCP with internal stenting was performed one day later. In another 4 patients with lateral duodenal wall perforations, the perforation was detected right after finishing ERCP and no further intervention was needed after closure in one patient. Biliary cannulation, endoscopic sphincterotomy, and stone removal were continued immediately after closure of perforation in another patient. In the last two patients, after an endoscopic closure, the second ERCP was successfully repeated during subsequent admission. There was no mortality in this study.

## 4. Discussion

Ovesco clip has been commercially available in Thailand since 2012. The main indication in Thailand is for a closure of iatrogenic perforation. We collected data from 3 tertiary-care hospitals in Thailand; 2 hospitals are in Bangkok and 1 hospital is in Songkhla province. There were 6 iatrogenic perforations that underwent immediate endoscopic closure by using Ovesco clips. The technical and clinical success rates were 100% and 83.3%, respectively. The success rates per locations were 100% in colon and 80% in duodenum. There was no mortality in this study. The overall success rate and the success rate for colonic and duodenal closure are in line with previous reports, 87.8% [7], 76.9% [13, 14], and 88.2% [7], respectively.

Clinical failure in one patient was due to a large, 40 mm, perforation. In this patient, the 2 Ovesco clips were dislodged after band ligation. Consequently, surgical intervention was performed but only to remove the Ovesco clips. In retrospect, the perforation was already closed endoscopically. The patient can be safely discharged 1 day after surgery.

There was no report on the upper limit of perforation size closure in human [7, 13, 14]. In nonsurvival porcine model, the success rate for closure of colonic wall defect sizes of 24–27 mm and 29–55 mm was 83% and 29%, respectively [15]. In a prospective human study, the clinical success rate on Ovesco closure of 30 mm defect was reported as 97% [12]. Our study demonstrates that all perforations with the size range from 10 to 15 mm can be successfully closed by one Ovesco clip, and only the 40 mm perforation failed to be closed by one Ovesco clip. In our experience, the optimal upper limit of perforation size to be closed by one Ovesco clip should be smaller than 30 mm.

In 4 patients in which perforation developed before finishing therapeutic procedures, Ovesco clip closure allowed the continuation of the original therapeutic plan immediately after Ovesco clip closure in 1 patient (25%), in the next day in 1 patient (25%), and in the next admission in 2 patients (50%). In our series, the primary plan of endoscopic treatment was achieved without the need for readmission in 50% of patients.

In conclusion, Ovesco clip is a promising device for an endoscopic closure of iatrogenic perforation as it provides a high success rate and low complication rate. The limitations of this study are its retrospective descriptive design and small sample size, and all closures are performed by expert endoscopists. The data from a comparative study with long-term follow-up is needed before recommending the Ovesco clip as the primary device for perforation treatment.

## Figures and Tables

**Table 1:** Patients characteristics and treatment of perforation.

Age/gender	Diagnosis	Cause of perforation	Size (mm)	Location	Treatment	NPO/LOS (days)
78/F	Bowel habit change	Colonoscope	10	Rectosigmoid junction	1 OTSC	1/3
56/F	Distal cholangiocarcinoma failed biliary cannulation	Needle-knife dilation of choledochoduodenostomy	15	Medial duodenal wall	1 OTSC, PTBD	10/22
70/F	Choledocholithiasis	Duodenoscope	10	Lateral duodenal wall	1 OTSC, ERCP with stone removal	7/15
60/M	Choledocholithiasis	Duodenoscope	13	Lateral duodenal wall	1 OTSC	1/2
58/F	Choledocholithiasis	Duodenoscope	13	Lateral duodenal wall	1 OTSC	3/8
39/M	Chronic pancreatitis with pancreatic duct stone and stricture after pancreatic stenting	Duodenoscope	40	Lateral duodenal wall	2 OTSCs with 1 band ligation	8/12

NPO, nil per oral; LOS, length of stay; OTSC, over-the-scope clip.

## Additional Points

Iatrogenic perforation traditionally requires surgery. Ovesco clip is a device that provides full thickness closure of gastrointestinal wall and has been increasingly used for a closure of iatrogenic perforation. It can be applied immediately in endoscopy suite and this in turn can prevent further spillage of gastrointestinal content into peritoneal space. It provides a high success rate especially in a lesion smaller than 30 mm and should be the preferred method for perforation closure.
